# Scleroderma renal crisis with coexisting segmental arterial mediolysis presenting as intraperitoneal bleeding: a case report

**DOI:** 10.1186/s13256-019-1993-z

**Published:** 2019-03-20

**Authors:** Shohei Kaneko, Eri Watanabe, Mai Abe, Shinji Watanabe, Hiroki Yabe, Shigehiro Kojima, Kenji Takagi, Keiji Hirai, Yoshiyuki Morishita, Chihiro Terai

**Affiliations:** 1 Department of Rheumatology, Saitama Medical Center, Jichi Medical University, 1-847 Amanuma-cho, Omiya-ku, Saitama, 330-8503 Japan; 2 Department of Nephrology, Saitama Medical Center, Jichi Medical University, 1-847 Amanuma-cho, Omiya-ku, Saitama, 330-8503 Japan; 3 Department of Surgery, Sainokuni Higashiomiya Medical Center, 1522 Toro-cho, Kita-ku, Saitama, 331-8577 Japan; 4 Department of Rheumatology, Sainokuni Higashiomiya Medical Center, 1522 Toro-cho, Kita-ku, Saitama, 331-8577 Japan

**Keywords:** Gastroepiploic artery aneurysm, Hypertension, Prednisolone, Renal dysfunction, Systemic sclerosis, Vascular disease

## Abstract

**Background:**

Segmental arterial mediolysis is a rare nonarteriosclerotic and noninflammatory vascular disease that may cause intraperitoneal bleeding. Scleroderma renal crisis is a rare complication of systemic sclerosis, leading to severe hypertension and renal dysfunction. To the best of our knowledge, this is the first reported case of a patient with concurrent systemic sclerosis with scleroderma renal crisis and pathologically confirmed segmental arterial mediolysis.

**Case presentation:**

We report a case of a 68-year-old Chinese woman diagnosed with systemic sclerosis who was found to have coexisting segmental arterial mediolysis. She presented with back pain, and massive intraperitoneal bleeding was detected by computed tomography. She underwent laparotomy, and the bleeding was found to originate from the gastroepiploic artery. The pathological examination demonstrated gastroepiploic arterial dissection caused by segmental arterial mediolysis. After surgery, she developed severe hypertension with hyperreninemia and progressive renal dysfunction. Given the risk factors of corticosteroid administration and the presence of anti-ribonucleic acid polymerase III antibody, she was diagnosed with scleroderma renal crisis. The patient was proved to have a very rare case of coexisting scleroderma renal crisis and segmental arterial mediolysis.

**Conclusions:**

There is no known etiological connection between segmental arterial mediolysis and systemic sclerosis or scleroderma renal crisis, but it is possible that coexisting segmental arterial mediolysis and scleroderma renal crisis may have interacted to trigger the development of the other in our patient.

## Background

Segmental arterial mediolysis (SAM) is a rare nonarteriosclerotic and noninflammatory vascular disease first described by Slavin *et al*. in 1976 [1]. SAM often develops in the abdominal visceral arteries, where it forms an aneurysm and causes intraperitoneal bleeding. Emergency surgery or transcatheter arterial embolization are required to treat bleeding due to rupture of SAM [2, 3]. Scleroderma renal crisis (SRC) is a severe and rare complication of systemic sclerosis (SSc), leading to severe hypertension and progressive renal dysfunction [4, 5]. Several risk factors have been associated the onset of SRC, including diffuse skin involvement, disease duration < 4 years, presence of anti-ribonucleic acid (RNA) polymerase III antibodies, and treatment with prednisolone (PSL) ≥ 15 mg/day within the previous 3 months [4, 6–9]. We report a case of a patient with SRC with several risk factors, coincidentally complicated with SAM, presenting as intraperitoneal bleeding.

## Case presentation

**Fig. 1:**
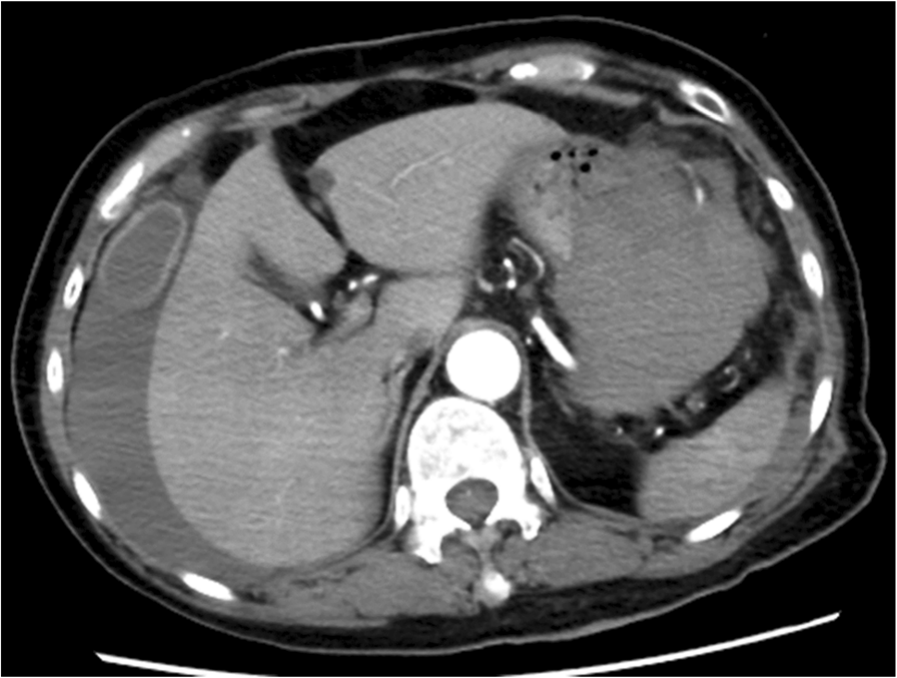
Contrast-enhanced computed tomography of the abdomen indicated massive intraperitoneal bleeding

In June, a 68-year-old Chinese woman with severe hypertension and renal dysfunction was admitted to our hospital for further treatment. The patient had no remarkable medical history or family history. The patient had noticed puffy swollen fingers and leg edema 1 year previously, as well as the gradual development of skin thickening and Raynaud phenomenon. She had been diagnosed with SSc by a local doctor and had been prescribed PSL 20 mg/day 25 days before admission. At 11 days before the current admission, the patient had visited the previous hospital with acute-onset chest and back pain with blood pressure 101/51 mmHg and pulse rate 111 beats/min, and contrast-enhanced computed tomography (CT) showed massive intraperitoneal bleeding (Fig. 1). At this time, contrast-enhanced CT showed normal findings of renal arteries, and laboratory findings showed serum creatinine (Cre) 1.1 mg/dl and blood urea nitrogen (BUN) 37.4 mg/dl. The patient accordingly underwent emergency laparotomy, and the bleeding from the gastroepiploic artery was stopped surgically. After surgery, she developed severe hypertension (260/129 mmHg) and progressive renal dysfunction (Cre, 6.4 mg/dl; BUN, 125.3 mg/dl). She was transferred to our hospital for further treatment.

On admission, physical examination showed bilateral lower limb edema, trismus, nail fold telangiectasia, and diffuse skin thickening. The patient’s modified Rodnan total skin thickness score was 33 points. No lung lesions were detected on chest CT. Laboratory findings (Table 1) showed normocytic, normochromic anemia and severe renal dysfunction with elevated levels of plasma renin activity at ≥ 20 ng/ml/h (normal, 0.3–2.9 ng/ml/h) and plasma aldosterone concentration 433 pg/ml (normal, 35.7–240 pg/ml). Immunological tests revealed positive antinuclear antibody and anti-RNA polymerase III antibody. Echocardiography showed mild left ventricular hypertrophy, a small amount of pericardial effusion, preserved ejection fraction of 80%, and no pulmonary hypertension. Renal artery ultrasound examination showed no evidence of renal artery stenosis.

**Table 1:** Laboratory data on admission

Laboratory test	Result
Complete blood count	
White blood cells	16,650/μl
Neutrophils	91%
Red blood cells	264 × 10^6^/μl
Hemoglobin	8.2 g/dl
Hematocrit	25.9%
Mean corpuscular volume	98 fl
Mean corpuscular hemoglobin	31 pg
Platelet	17.9 × 10^4^/μl
Coagulation	
PT-INR	1.01
APTT	31 s
Biochemistry	
Total protein	5 g/dl
Albumin	2.9 g/dl
Total bilirubin	0.49 mg/dl
Aspartate aminotransferase	32 U/L
Alanine aminotransferase	47 U/L
Lactate dehydrogenase	636 U/L
C-reactive protein	0.63 mg/dl
Sodium	133 mEq/L
Potassium	4.9 mEq/L
Blood urea nitrogen	127 mg/dl
Creatinine	6.6 mg/dl
Estimated glomerular filtration rate	5.4 ml/min/1.73 m^2^
Brain natriuretic peptide	4566 pg/ml
Plasma renin activity	≥ 20 ng/ml/h
Plasma aldosterone concentration	433 pg/ml
Autoantibody test	
Antinuclear antibody	320 index (speckled pattern)
Anti-RNA polymerase III antibody	≥150
Anti-topoisomerase I antibody	Negative
Anti-centromere antibody	Negative
Anti-RNP antibody	Negative
Anti-SSA antibody	Negative
Anti-SSB antibody	Negative
PR3-ANCA	< 1.0 IU/ml
MPO-ANCA	1.1 IU/ml
Urinalysis	
pH	5
Specific gravity	1.012
Protein	2+
Glucose	–
Occult blood	3+
Red blood cells	1–4/LPF
White blood cells	Countless/HPF
Epithelial cell casts	1–4/HPF
Granular casts	1–4/LPF
Waxy casts	1–4/whole field
NAG	20 U/L
β_2_-Microglobulin	321 μg/L
24-h urine collection	
Urinary volume	290 ml/day
Creatinine clearance	1.3 ml/min
Protein	354 mg/day
Glucose	0.03 g/day

*Abbreviations*: *PT-INR* prothrombin time-international normalized ratio, *APTT* activated partial thromboplastin time, *anti-RNA polymerase III antibody* anti-ribonucleic acid polymerase III antibody, *anti-RNP antibody* anti-ribonucleoprotein antibody, *anti-SSA antibody* anti-Sjögren’s syndrome-related antigen A antibody, *anti-SSB antibody* anti-Sjögren’s syndrome-related antigen B antibody, *PR3-ANCA* proteinase-3-antineutrophil cytoplasmic antibody, *MPO-ANCA* myeloperoxidase-antineutrophil cytoplasmic antibody, *LPF* low-power field, *HPF* high-power field, *NAG* N-acetyl-β-D-glucosaminidase

The patient was diagnosed with SSc and developed severe hypertension and progressive renal dysfunction with proteinuria and hematuria, thus fulfilling the criteria for SRC [5, 7, 10, 11]. The patient also had several risk factors for SRC (duration of disease < 4 years, diffuse cutaneous form, administration of PSL ≥ 15 mg/day, and anti-RNA polymerase III antibody) [7–9]. No obvious thrombocytopenia, schistocytes, or elevation of direct bilirubin was present; hence, we had little evidence to diagnosis the patient with complications of thrombotic microangiopathy.

**Fig. 2:**
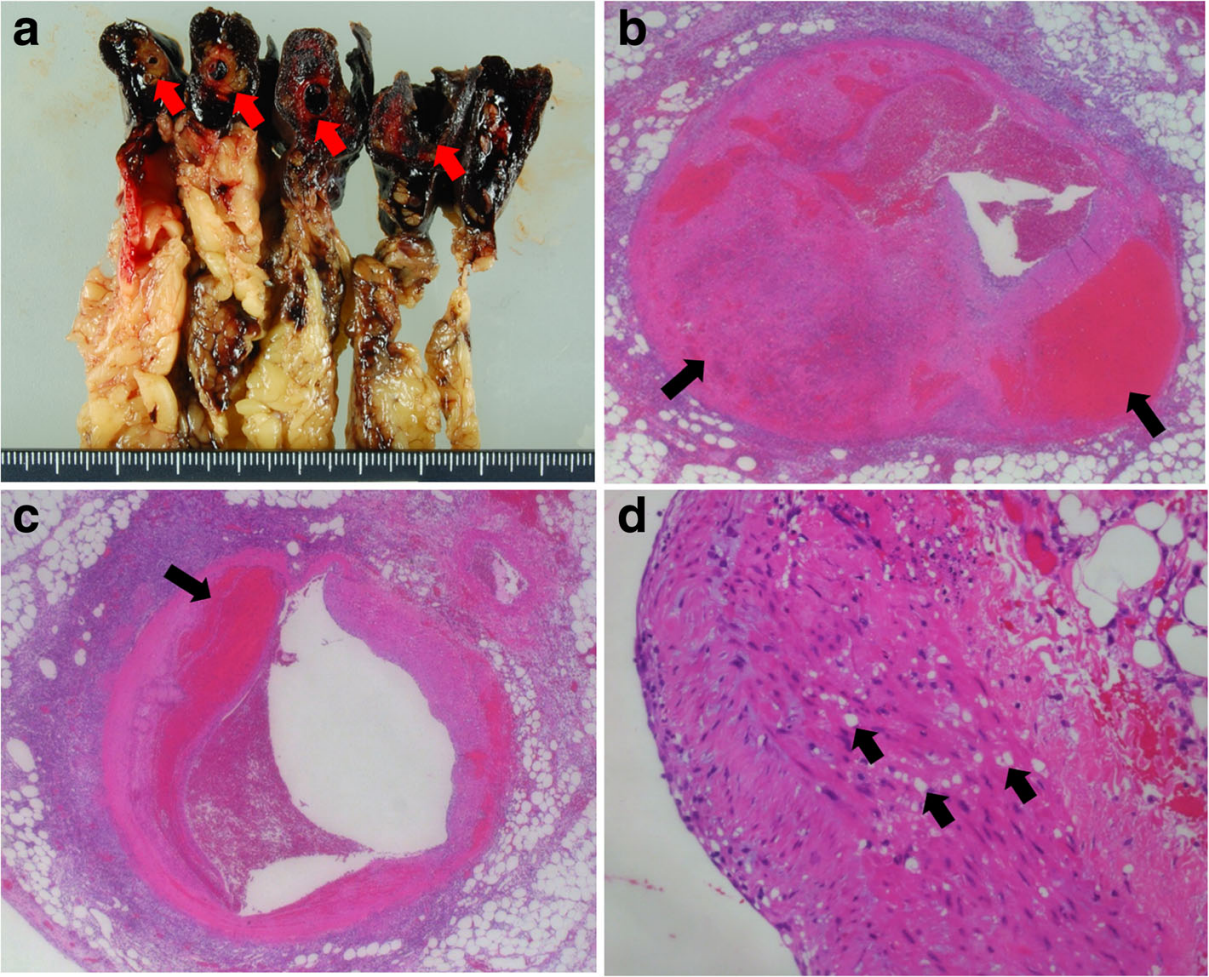
Pathological findings of the gastroepiploic artery. Macroscopic specimen of gastroepiploic artery (*red arrow*) with greater omentum (**a**). The gastroepiploic artery was surrounded by hematoma. The arterial wall was dissected (*black arrow*), and the dissected cavity was filled with hematoma and fibrin (**b** and **c**; H&E stain; original magnification, 20×). Multiple vacuoles (*black arrow*) were displayed in the medial muscle layer (**d**; H&E stain; original magnification, 40×)

The pathological examination of excised gastroepiploic artery at the previous hospital showed the dissection at the media–adventitia junction, and the dissected cavity was filled with hematoma, indicating gastroepiploic artery aneurysm (Fig. 2a–c). In addition, multiple vacuoles were revealed in the medial muscle layer (Fig. 2d), which was characteristic of SAM [1, 12]. We diagnosed a gastroepiploic artery aneurysm caused by SAM, with intraperitoneal bleeding due to rupture of the aneurysm. This patient was therefore confirmed as having a very rare case of coexisting SSc with SRC and SAM.

From the first day of admission, she received the angiotensin-converting enzyme inhibitor (ACE-I) enalapril at 5 mg/day, and maintenance hemodialysis was started. PSL was gradually reduced to 2.5 mg/day because of its increased risk of SRC. Her plasma aldosterone concentration decreased gradually (to 75.6 pg/ml on hospital day 45); however, the control of blood pressure was insufficient, and use of a combination of multiple antihypertensive drugs was required. Eventually, maintenance antihypertensive therapy included enalapril 5 mg/day, olmesartan 40 mg/day, and nifedipine 80 mg/day. Despite these treatments, her renal dysfunction did not improve at all. She was discharged on hospital day 57 and continued maintenance hemodialysis and blood pressure control thereafter. At the 1-year follow-up visit, she remained on dialysis, and her blood pressure was well-controlled with enalapril 2.5 mg/day and nifedipine 10 mg/day.

## Discussion and conclusions

SAM is a rare vasculopathy characterized by nonarteriosclerotic and noninflammatory vacuolar degeneration of smooth muscle cells of the arterial media, melting of the media, and gap formation. It was reported by Slavin *et al*. in 1976, but its etiology remains unknown to date [1]. Disruption of the intima allows the remaining adventitia to expand, leading to aneurysm formation [13]. SAM frequently occurs in the abdominal visceral arteries. Inada *et al*. collected 28 cases of SAM appearing on the abdominal visceral artery and reported that 50% of SAM occurred in the middle colonic artery, 21% in the gastric artery, and 18% in the gastroepiploic artery [2]. Pathological examination is necessary to make a definite diagnosis, but it is difficult to perform because of invasiveness. Our patient demonstrated typical pathological findings of multiple vacuoles in the medial muscle layer, leading to a definite diagnosis. As a category of nonarteriosclerotic and noninflammatory vascular disease, fibromuscular dysplasia and cystic medial necrosis are important differential diagnoses [14–16]. The common histological feature of fibromuscular dysplasia is medial fibrodysplasia. The collagenized regions alternate with thinned areas of media typically [14]. Clinical features of fibromuscular dysplasia are present in young females and have a predisposition for the medium-sized arteries, especially the renal artery [17]. Cystic medial necrosis occurs in the aorta and large vessels of patients with Marfan syndrome [18] and causes arterial dissection due to disruption of the medial elastic fiber based on congenital fragility of elastic fiber [14]. These clinical and histological features of fibromuscular dysplasia and cystic medial necrosis can be distinguished from SAM. Management of SAM involves embolization, stenting, or surgical resection of the injured arteries [2, 3], though SAM has also been reported to disappear naturally in some cases [19].

SRC is a rare and life-threatening complication of SSc. Without appropriate treatment, it results in renal death and high mortality [5]. SRC is characterized by malignant hypertension and acute renal failure. Although its pathogenesis is incompletely understood [20], it has been considered that proliferation of renal vascular intima [21] and renal vasospasm (so-called “renal Raynaud”) [22] may cause decreased renal blood flow. In addition, hypotension caused by infections, drugs, or dehydration may also contribute to the decreased renal blood flow [23]. Decreased renal blood flow leads to activation of the renin-angiotensin system and the development of severe hypertension. Steen *et al*. identified several risk factors associated with SRC, including diffuse skin involvement, disease duration < 4 years, a recent cardiac event, recent onset of anemia, presence of anti-RNA polymerase III antibodies, and treatment with PSL ≥ 15 mg/day or cyclosporine within the previous 3 months [4, 6–9]. Our patient was thus at high risk of developing SRC because of diffuse cutaneous SSc, a disease duration of 1 year, anti-RNA polymerase III antibody positivity, and the administration of PSL 20 mg/day from 25 days before admission. She was prescribed ACE-I and angiotensin II receptor blockers, whose effectiveness has been established in the treatment of SRC [7, 10], but her renal outcome was poor. Steen *et al*. reported that poor prognosis factors associated with SRC were male sex, older age, presence of congestive heart failure, Cre level > 3 mg/dl at the initiation of treatment, and more than 3 days needed to control blood pressure [10, 24]. Our patient had three of these factors: older age, severe renal dysfunction (Cre 6.4 mg/dl) at the initiation of treatment, and > 3 days required to regulate blood pressure; thus, we believed it might be difficult to rescue her renal function.

To the best of our knowledge, no previous reports have described the obvious relationship between SAM and connective tissue diseases, including SSc and SRC. Therefore, our patient had a very rare condition of coincidental coexisting SAM and SRC. Although these are independent diseases, it is possible that they may have interacted to trigger the development of the other in our patient. We considered two possible mechanisms to explain the development of SRC and the aneurysmal rupture of SAM. First, the SRC may have preceded the onset of aneurysmal rupture of SAM, and the SRC-related hypertension may have triggered the aneurysmal rupture, resulting in intraperitoneal bleeding. Her blood pressure was not elevated before surgery, but SRC-related hypertension may have been masked by hemorrhagic hypotension due to the intraperitoneal bleeding. Second, hemodynamic change due to hemorrhagic hypotension or surgical stress may have contributed to the decreased renal blood flow and may have led to activation of the renin-angiotensin system and the development of SRC. These theories are only hypotheses, but the simultaneous occurrence of SAM and SRC certainly exacerbated the patient’s condition.

In conclusion, both SRC and SAM are uncommon but life-threatening clinical conditions with delayed treatment. We report a very rare case of SSc with SRC coexisting with SAM and presenting with intraperitoneal bleeding. There is no known etiological connection between SAM and SSc or SRC, but it is possible that coexisting SAM and SRC may have interacted to trigger the development of the other in our patient.

## Abbreviations

ACE-I: Angiotensin-converting enzyme inhibitor
BUN: Blood urea nitrogen
Cre: Creatinine
CT: Computed tomography
PSL: Prednisolone
SAM: Segmental arterial mediolysis
SRC: Scleroderma renal crisis
SSc: Systemic sclerosis
